# Open and Arthroscopic Acetabular Labral Reconstructions Are Associated With Improvements in Hip Patient‐Reported Outcome Measures at 1 Year, but Only an Open Approach Is Associated With Significant Reductions in Pain

**DOI:** 10.1002/ars2.70003

**Published:** 2026-07-29

**Authors:** John R. Baumann, Kylee Rucinski, James L. Cook, Brett D. Crist, Cory R. Crecelius, Steven F. DeFroda

**Affiliations:** ^1^ Thompson Laboratory for Regenerative Orthopaedics Hip Preservation Center Missouri Orthopaedic Institute Department of Orthopaedic Surgery University of Missouri Columbia Missouri U.S.A.

## Abstract

**Purpose:**

To compare initial outcomes for open versus arthroscopic acetabular labral reconstruction (ALR) using fresh meniscus allograft transplantation.

**Methods:**

With informed consent, patients scheduled to undergo ALR were enrolled into a registry. Patients included in this study underwent fresh meniscus allograft transplantation for irreparable acetabular labrum deficiency with a minimum of 1 year of complete follow‐up data. Patients were excluded if they were pregnant, were incarcerated, or underwent previous or concurrent femoral head cartilage repair procedures. Patient‐reported outcome measures were collected preoperatively and 3 months, 6 months, and 1 year postoperatively. Fisher's exact and Wilcoxon signed‐rank tests were used for statistical analysis, with *P* < .05 considered statistically significant.

**Results:**

A total of 32 consecutive patients met inclusion criteria and were analyzed (22 open, 10 arthroscopic). Overall, 22 open labral reconstructions (100%) were deemed successful, and 9 arthroscopic labral reconstructions (90%) were deemed successful. There was no statistically significant difference in initial failure rates. Both cohorts had significant improvements in Hip Disability and Osteoarthritis Outcome for Joint Replacement, Patient‐Reported Outcomes Measurement Information System (PROMIS) Physical Function, and PROMIS Physical Health from the preoperative to 1‐year postoperative timepoints. For the arthroscopic ALR cohort, there was no significant difference in preoperative and postoperative Visual Analog Scale (6.2 vs 4.6) or PROMIS Pain Interference scores (65.5 vs 58.4). Preoperative PROMIS Pain Interference was significantly higher in the arthroscopic cohort compared with the open cohort (65.5 vs 61.7). Three months (4.7 vs 2.8), 6 months (4.1 vs 2.4), and 1 year (4.6 vs 1.9) Visual Analog Scale pain scores were significantly higher for the arthroscopic cohort compared with the open cohort. At 1 year postoperatively, the minimum clinically important difference threshold was reached for Hip Disability and Osteoarthritis Outcome for Joint Replacement in the arthroscopic cohort and for Visual Analog Scale pain score and Hip Disability and Osteoarthritis Outcome for Joint Replacement in the open cohort.

**Conclusions:**

The results of this study suggest that open and arthroscopic ALRs with fresh meniscus allograft may significantly improve patient‐reported outcomes associated with hip function, health, and recovery at 1 year postoperatively. The open cohort, but not the arthroscopic cohort, had significant improvements in pain at 1 year postoperatively.

**Level of Evidence:**

Level III, retrospective comparative study.

## Graphical abstract

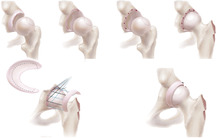

Acetabular labrum pathology impacts a substantial number of patients. Trauma, overuse injuries, and femoroacetabular impingement frequently lead to labral pathology that, if left untreated, can progress to hip osteoarthritis. A fully functional labrum is essential for maintaining hip joint health, as it plays crucial roles in joint stability, sustaining the suction‐seal effect, distributing loads, and protecting the articular cartilage.^1^, ^2^, ^3^, ^4^, ^5^, ^6^, ^7^, ^8^, ^9^ These vital functions rely on the labrum's cellular activity, extracellular matrix, and material properties. Therefore, any repair or reconstruction of a deficient acetabular labrum must aim to restore these functional components that define this specialized fibrocartilage to achieve optimal hip preservation. While labral repair is preferred when possible, many labral defects are irreparable, necessitating acetabular labral reconstruction (ALR) to restore joint function and preserve joint health.^10^, ^11^, ^12^

There are several different autograft or allograft options available for ALR. Common autograft options include the ligamentum teres, joint capsule, rectus femoris, fascia lata, iliotibial band, gracilis tendon, and quadriceps tendon. Commonly used allografts include the anterior and posterior tibialis tendons, semitendinosus, peroneus brevis tendon, and fascia lata.^10^, ^13^, ^14^, ^15^, ^16^, ^17^ In the United States of America, allografts are more frequently used than autografts due to their availability, the avoidance of donor site morbidity, and reduced anesthesia and operative times.^10^, ^18^, ^19^, ^20^, ^21^, ^22^, ^23^ Fresh‐frozen tendon allografts are the predominant choice for ALR in the USA and have shown acceptable outcomes. However, tendon allograft ALR failure rates can reach approximately 25%, potentially due to suboptimal properties of frozen tendons for restoration of the functional components of labral fibrocartilage.^24^, ^25^, ^26^, ^27^, ^28^, ^29^

Previous research by this group suggests that ALR with allografts should be prioritized over labral resection for treating irreparable acetabular labrum deficiencies. Fresh meniscus allograft transplantations have been associated with clinically relevant mechanistic outcome measures that are superior to those associated with fresh‐frozen tendon allograft transplants.^30^ Consequently, we have implemented an evidence‐based shift in practice to the use of fresh meniscus allografts for the treatment of patients indicated for ALR at our institution.^31^, ^32^

ALR can be performed using an open or arthroscopic approach. Ongoing research at this institution and many others aims to optimize surgical technique and graft choice for ALR. Recently, open and arthroscopic approaches have been used at our institution when performing fresh meniscal allograft ALRs. Published research comparing outcomes following open versus arthroscopic ALR is limited. Therefore, the purpose of this study was to compare initial outcomes for open versus arthroscopic ALR using fresh meniscus allograft transplantation. The study was designed to test the null hypothesis that there would be no significant differences in patient‐reported outcome measures (PROMs) or failure rates through the first year after ALR performed using fresh meniscus allograft transplants for patients treated with arthroscopic versus open approaches.

## METHODS

This study was approved and executed under #2003053 from the University of Missouri Institutional Review Board. With informed consent, patients scheduled to undergo ALR were enrolled into the IRB‐approved registry designed to monitor outcomes following hip preservation surgeries. After thorough preoperative evaluation and counseling, patients chose ALR using fresh meniscus allograft transplantation over other nonsurgical or surgical options and were preauthorized for the procedure by their health insurance carriers. The decision‐making and consent process included detailed discussions with the attending surgeon and our institution's joint preservation team.

Patients were included for analyses when they underwent fresh meniscus allograft transplantation for irreparable acetabular labrum deficiency with complete 1‐year follow‐up data available. Exclusion criteria for this study included patients who were pregnant, were incarcerated, or underwent previous or concurrent femoral head or acetabular cartilage repair procedures. Patients with concurrent or previous femoral head cartilage repair procedures were excluded as this procedure has only been offered through an open approach at our institution. Acetabular osteochondral allograft procedures are no longer offered at our institution due to poor outcomes.

### Fresh Meniscus Allograft Preservation

Allografts used in these procedures were recovered from eligible organ and tissue donors for processing, testing, and storage at an American Association of Tissue Banks–accredited tissue bank (MTF Biologics, Edison, NJ). Tissues were preserved using the Missouri Osteochondral Preservation System, which has been validated to maintain significantly higher viable cell density at the time of transplantation compared with other methods used for osteochondral and meniscal tissue preservation.^31^, ^33^, ^34^, ^35^ Once all required safety testing was completed, meniscus allografts were used for ALR in compliance with the US Food and Drug Administration's classification of human cell and tissue products under Section 361 of the Public Health Service Act within 56 days from recovery.

### Open Surgical Procedure

**FIGURE 1:**
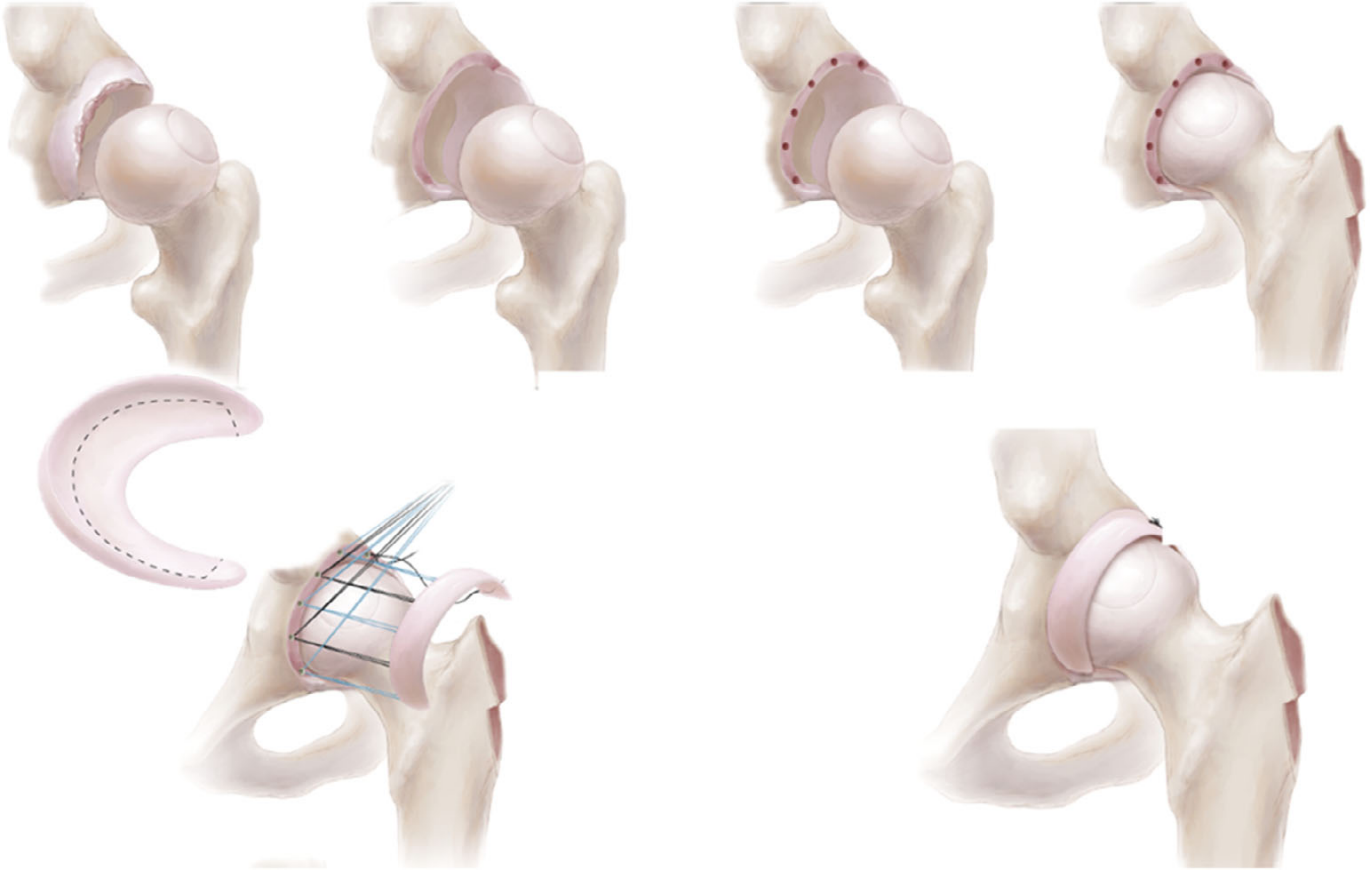
Open acetabular labral reconstruction with fresh meniscus allograft. Illustration of the left hip depicting the steps of the open acetabular labral reconstruction with fresh meniscus allograft. Illustrations depict trochanteric osteotomy, resection of irreparable labral tissues, preparation of the recipient bed, suture placement in 1‐cm intervals from anterior to posterior, and final fixation.

All open ALRs were performed by senior authors (B.C., S.D.) via surgical hip dislocation.^36^ A step‐cut greater trochanteric osteotomy was performed (Figure 1).^37^ Irreparable labral tissue was resected and the defect size was measured. The recipient bed was prepared using a high‐speed burr to decorticate to bleeding bone. Meniscal allografts were prepared by sharp dissection followed by trimming to match the size of native, remaining labrum. Passing sutures were placed along the peripheral margin of the meniscus allograft at 1‐cm intervals such that each entered and exited at the inferior and superior margins, engaging meniscal circumferential fibers. Knotless labral‐based suture anchors and a posterior junctional suture anchor were placed at 1‐cm intervals from anterior to posterior along the recipient bed while the graft was being prepared. The hip was then reduced, and all anchor sutures were passed into the meniscus allograft using the preplaced passing sutures. Sequential tightening of the anchor sutures, from anterior to posterior, secured the allograft to the acetabular recipient bed. Finally, the posterior junctional anchor suture was used to attach the allograft to the remaining native labrum, followed by tightening all sutures for complete seating of the meniscus allograft.

### Arthroscopic Surgical Procedure

**FIGURE 2:**
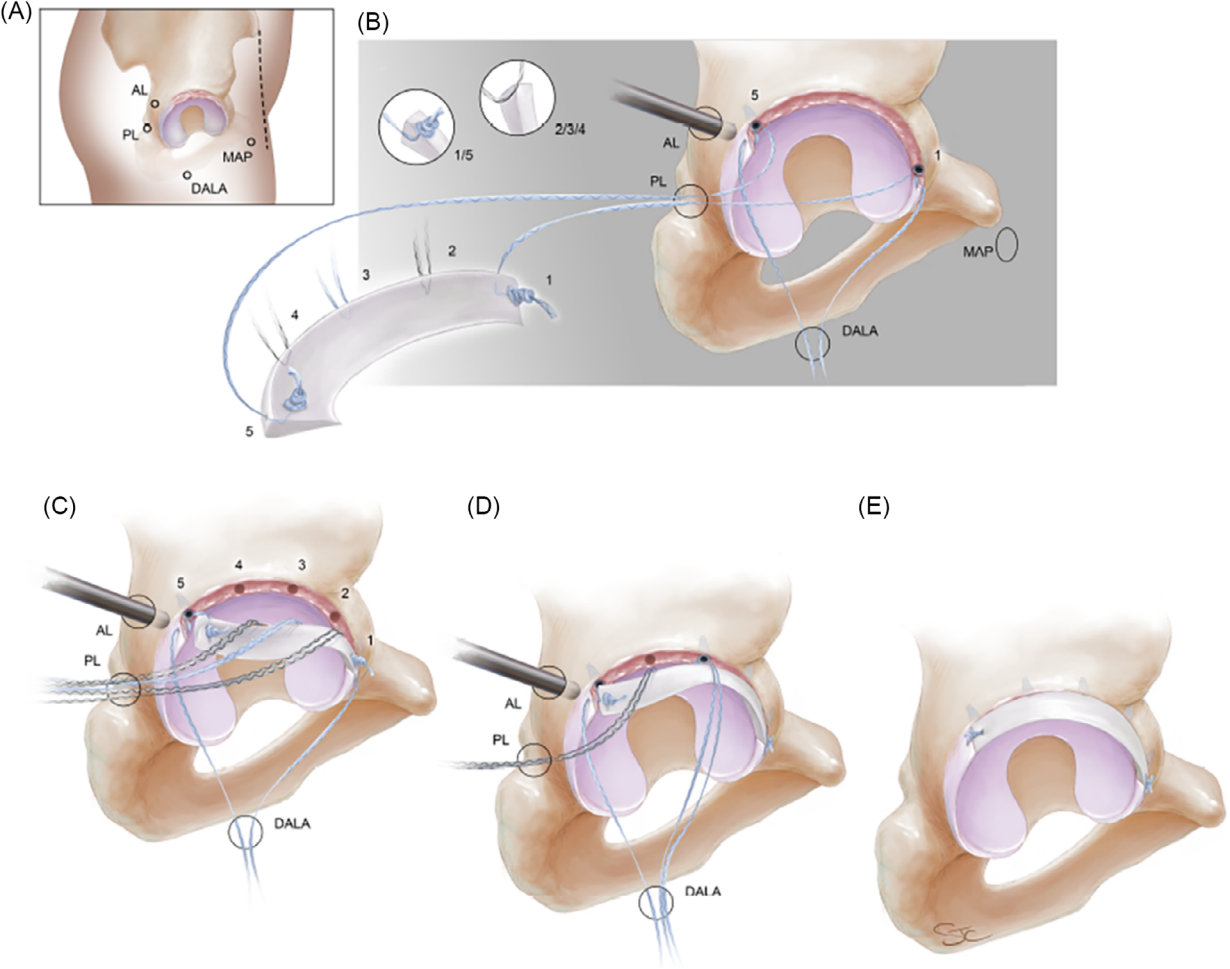
Arthroscopic acetabular labrum reconstruction with fresh meniscus allograft. Illustration of the right hip depicting the steps of the arthroscopic acetabular labral reconstruction with fresh meniscus allograft. (A) Lateral view of the hip showing portal placement. (B) Depiction of the acetabular labrum outlining camera placement, suture management, and graft shuttling. (C‐E) Depiction of the acetabular labrum showing stepwise fixation from anterior (C) to posterior (D) and final fixation (E). (AL, anterolateral portal; DALA, direct anterolateral portal; MAP, modified midanterior portal; PL, posterolateral portal.) Image originally published in Beyer JJ, Richards JA, Woodard DR, Cook JL, Crist BD, DeFroda SF. Arthroscopic hip labral reconstruction with fresh meniscal allograft. *Arthrosc Tech*. 2025;14(5):103459. doi:10.1016/j.eats.2025.103459.

All arthroscopic ALRs were performed by senior authors (B.C., S.D.) (Figure 2). Anterolateral, modified midanterior, distal anterolateral, anterior proximal medial, and posterior portals were used to perform the procedure. Traction was applied to distract the hip joint. The remaining labral tissue was identified, and nonviable‐appearing labral tissue was debrided. Using a burr, the acetabular rim was prepared for labral transplantation. An anchor was placed at the lateral and medial extent of the labral deficiency. The size of the defect was measured. Pilot holes for knotless anchors were drilled. Meniscal allografts were prepared by sharp dissection followed by trimming to match the defect length and the width/height of native, remaining labrum. The meniscus allograft was delivered into the joint through the posterior portal using the Kite technique.^38^ One suture from the anterior/medial‐most and 1 suture from the posterior/lateral‐most anchor were retrieved. The anterior‐most and posterior shuttle stitches were passed through the meniscus. The sutures through the meniscus body were retrieved via the accessory proximal medial portal. A knotted anchor suture was tied anteriorly. Next, the sutures within the meniscus were retrieved via the distal anterolateral portal and placed into knotless suture anchors tensioned sequentially to allow for good fixation and contouring of the meniscal allograft. Lastly, a posterior knotted anchor was used to fix the meniscus allograft. Traction was released and there was a restoration of the suction seal. Capsular plication of the capsulotomy was performed with a suture shuttling device.^14^

### Rehabilitation Protocols

Each patient was provided with tailored postoperative rehabilitation instructions, delivered both verbally and in written form. These instructions were also shared with outpatient physical therapists involved in the patient's recovery.

Postoperatively, arthroscopic ALR patients were restricted to 25 pounds of weight‐bearing for 2 weeks. Open ALR patients were restricted to 25 pounds of weight‐bearing for 6 weeks and prohibited from performing active hip abduction. After 2‐week clinical examination for the arthroscopic ALR patients and 6‐week clinical examination and radiographs for the arthroscopic ALR patients, weight‐bearing activities were gradually reintroduced. Patients in both cohorts were permitted to discontinue the use of crutches once cleared by their surgeon and after their gait is normal and pain free. Physical therapy was to begin within the first 2 weeks after surgery and continue for up to 6 months. For both cohorts, stationary bike use was allowed starting 1 week after surgery, and return to sports was permitted around 6 months postoperatively.

Patient adherence to the rehabilitation protocol was monitored through direct communication and reports from outpatient physical therapists.

### Outcome Measures

PROMs collected included Visual Analog Scale (VAS) for pain, Hip Disability and Osteoarthritis Outcome for Joint Replacement (HOOS‐JR), Patient‐Reported Outcomes Measurement Information System (PROMIS) Physical Function, PROMIS Physical Health, PROMIS Mental Health, and PROMIS Pain Interference. All PROMs were assessed preoperatively and at 3 months, 6 months, and 1 year after surgery. Demographic information, surgical details, postoperative complications, and reoperation data were extracted from patients’ electronic medical records. ALR treatment failure was defined as conversion to arthroplasty, while revision was characterized by reoperation to modify the previously implanted allograft in any way. Decisions regarding revision surgery or arthroplasty were made through discussions between the surgeon and patient, considering the failure mechanism, treatment options, prognosis, and patient expectations and preferences. Outcomes were considered successful when patients returned to functional activities without requiring revision or arthroplasty surgery. Final follow‐up (FFU) was defined as final in‐person visit with the orthopaedic team and/or final PROM completion timepoint.

### Statistical Analysis

Descriptive statistics were used to summarize means, ranges, and percentages. Fisher's exact tests were used to identify significant differences in proportions. For comparisons showing significant differences, odds ratios were calculated. Wilcoxon signed‐rank tests were used to compare outcome measures between cohorts at each timepoint. A significance level of *P* < .05 was considered statistically significant. Thresholds for minimum clinically important differences and ±1 standard deviation (SD) for PROMIS healthy population means were determined and reported for each cohort.^39^, ^40^, ^41^

## RESULTS

### Patient Population

A total of 32 consecutive patients met the inclusion criteria and were analyzed. There were 22 patients who underwent open labral reconstruction and 10 patients who underwent arthroscopic labral reconstruction (Table 1). The mean body mass index was significantly higher in the arthroscopic labral reconstruction cohort (mean = 30.4 ± 4.1) than in the open labral reconstruction cohort (mean = 27.0 ± 6.4) (*P* = .042). There were no significant differences in age (*P* = .839), sex (*P* = .387), nicotine use (*P* = 1.0), insurance type (*P* = .516), laterality (*P* = 1.0), or nonadherence (*P* = 1.0) between patients in the arthroscopic versus open cohorts. All fresh meniscus allografts used were confirmed to have >90% cell viability at the time of transplantation.^31^, ^32^, ^33^, ^34^, ^35^, ^42^, ^43^, ^44^, ^45^, ^46^, ^47^

**TABLE 1:** Patient Demographics by Cohort

	Open		Arthroscopic			*P* Value
No. of patients	22		10			
Sex	14 female (63.6%)	8 male (36.4%)	5 female (50%)		5 male (50%)	.387
Final follow‐up (mo)	38.8 (range: 14‐72)		18.2 (range: 12‐24)			**<.001**
Success at 1 yr	22 yes (100%)	0 no (0%)	9 yes (90%)		1 no (10%)	.313
Mean BMI	27.0 ± 6.4		30.4 ± 4.1			**.042**
Mean age	39.59 ± 10.1		30.75 ± 9.8			.839
Nicotine use	1 yes (4.5%)	21 no (95.5%)	0 yes (0%)		10 no (100%)	1.0
Laterality	7 left (31.8%)	15 right (68.2%)	3 left (30%)		7 right (70%)	1.0
Adherence	18 yes (81.8%)	4 no (18.2%)	8 yes (80%)		2 no (20%)	1.0
Insurance	18 commercial (81.8%)	4 Medicare/Medicaid (18.2%)	8 commercial (80%)	1 Medicare/Medicaid (10%)	1 military (10%)	.516

*Note*: Patient demographics are reported for respective cohorts. Percentages by cohort or ranges are represented in parenthesis when appropriate. Standard deviations are provided with cohort means when appropriate. *P* values are reported to compare cohorts when appropriate, with statistically significant *P* values in bold.

BMI, body mass index.

### Concurrent Procedures

A total of 31 patients (96.9%) underwent at least 1 concurrent procedure. The 22 open patients underwent a trochanteric osteotomy; 4 open patients also underwent concurrent proximal femoral rotational osteotomy. A total of 21 patients (17 open, 4 scope) underwent head and neck osteoplasty. Thirteen patients (8 open, 5 scope) underwent concurrent acetabuloplasty, 3 open patients underwent anterior inferior iliac spine decompression, and 5 patients (1 open, 4 scope) underwent concurrent hardware removal. Four patients (1 open, 3 scope) underwent staged proximal femoral rotational osteotomy (n = 1 open) or periacetabular osteotomy (n = 3 scope) within 1 month of their labral reconstruction. Concurrent proximal femoral rotational osteotomy (*P* = 1), head and neck osteoplasty (*P* = 1), acetabuloplasty (*P* = 1), hardware removal (*P* = 1), spine decompression (*P* = 1), and staged osteotomy (*P* = 1) were not significantly associated with failure risk.

### Patient‐Reported Outcome Measures

When comparing preoperative PROMs between cohorts (Table 2), there were no significant differences in HOOS‐JR (*P* = .802), VAS pain (*P* = .96), PROMIS Physical Function (*P* = .089), or PROMIS Mental Health (*P* = .852). Preoperative PROMIS Physical Health was significantly lower in the arthroscopic cohort compared with the open cohort (*P* = .013). Preoperative PROMIS Pain Interference was significantly higher in the arthroscopic cohort compared with the open cohort (*P* = .013).

**TABLE 2:** Patient‐Reported Outcome Measures by Cohort

	Preoperatively			3 Mo Postoperatively			6 Mo Postoperatively			1 Yr Postoperatively		
	Open	Arthroscopic	*P* Value	Open	Arthroscopic	*P* Value	Open	Arthroscopic	*P* Value	Open	Arthroscopic	*P* Value
**HOOS‐J**	56.9	54.9	.802	65.3	57.9	.116	73.5	67.6	.325	78.1	73.4	.429
*Std dev*	*12.3*	*12.0*		*12.3*	*14.8*		*14.7*	*12.8*		*15.3*	*7.3*	
**VAS pain score**	4.9	6.2	.96	2.8	4.7	**.015**	2.4	4.1	**.047**	1.9	4.6	**.022**
*Std dev*	*1.9*	*1.0*		*1.7*	*1.8*		*2.3*	*1.5*		*2.3*	*1.5*	
**PROMIS Physical Function**	41.0	37.6	.089	39.3	39.4	.815	43.4	40.0	.191	45.2	45.9	.928
*Std dev*	*6.8*	*3.3*		*6.4*	*5.1*		*7.0*	*3.4*		*7.1*	*5.2*	
**PROMIS Physical Health**	41.7	37.3	**.013**	42.1	40.5	.557	46.4	42.3	.192	47.6	44.5	.356
*Std dev*	*4.0*	*3.3*		*5.4*	*3.5*		*7.4*	*7.0*		*6.7*	*5.7*	
**PROMIS Mental Health**	49.7	50.6	.852	47.8	48.2	.971	49.8	47.4	.673	47.9	48.4	.910
*Std dev*	*8.3*	*6.7*		*9.5*	*6.9*		*10.1*	*8.1*		*7.6*	*8.6*	
**PROMIS Pain Interference**	61.7	65.5	**.013**	59.7	62.2	.238	57.7	61.7	.333	52.8	58.4	.310
*Std dev*	*5.4*	*3.3*		*6.3*	*4.6*		*7.6*	*7.5*		*8.0*	*9.2*	

*Note*: Patient‐reported outcomes were compared between treatment cohorts at the preoperative timepoint and the 3‐month, 6‐month, and 1‐year postoperative timepoints. Values reported represent means, with respective standard deviations italicized below. *P* values are reported to compare means between cohorts at each respective timepoint, with statistically significant *P* values in bold.

HOOS‐JR, Hip Disability and Osteoarthritis Outcome for Joint Replacement; PROMIS, Patient‐Reported Outcomes Measurement Information System; Std dev, standard deviation; VAS, Visual Analog Scale.

When comparing 3‐month postoperative PROMs, there were no significant differences in HOOS‐JR (*P* = .116), PROMIS Physical Function (*P* = .815), PROMIS Physical Health (*P* = .557), PROMIS Mental Health (*P* = .971), or PROMIS Pain Interference (*P* = .238). Three‐month VAS pain scores were significantly higher for the arthroscopic cohort compared with the open cohort (*P* = .015).

When comparing 6‐month postoperative PROMs, there were no significant differences in HOOS‐JR (*P* = .325), PROMIS Physical Function (*P* = .191), PROMIS Physical Health (*P* = .192), PROMIS Mental Health (*P* = .673), or PROMIS Pain Interference (*P* = .333). Six‐month VAS pain scores were significantly higher for the arthroscopic cohort compared with the open cohort (*P* = .047).

When comparing 1‐year postoperative PROMs, there were no significant differences in HOOS‐JR (*P* = .429), PROMIS Physical Function (*P* = .928), PROMIS Physical Health (*P* = .356), PROMIS Mental Health (*P* = .910), or PROMIS Pain Interference (*P* = .310). One‐year VAS pain scores were significantly higher for the arthroscopic cohort compared with the open cohort (*P* = .022).

When comparing preoperative and 1‐year postoperative outcome measures for the open meniscus transplantation cohort, there were significant improvements in HOOS‐JR (*P* = .0012), VAS pain (*P* = .0006), PROMIS Physical Function (*P* = .0301), PROMIS Physical Health (*P* = .005), and PROMIS Pain Interference (*P* = .01). There was no significant difference in preoperative and 1‐year postoperative PROMIS Mental Health (*P* = .744) in the open cohort.

When comparing preoperative and 1‐year postoperative outcome measures for the arthroscopic meniscus transplantation cohort, there were significant improvements in HOOS‐JR (*P* = .05), PROMIS Physical Function (*P* = .015), and PROMIS Physical Health (*P* = .02). There were no significant differences in preoperative and postoperative VAS pain (*P* = .3081), PROMIS Mental Health scores (*P* = .5735), and PROMIS Pain Interference (*P* = .2219) in the arthroscopic cohort.

In the arthroscopic cohort, the minimum clinically important difference threshold was reached for HOOS‐JR at 1 year of follow‐up, and follow‐up PROMIS scores fell within 1 SD of the healthy population means as follows: PROMIS PF (1 year), PROMIS PH (1 year), and PROMIS MH (3 months, 6 months, and 1 year). In the open cohort, minimum clinically important difference thresholds were reached for HOOS‐JR at 6 months and maintained through 1 year of follow‐up and for VAS pain score at 3 months and maintained through 1 year of follow‐up; follow‐up PROMIS scores fell within 1 SD of the healthy population means as follows: PROMIS PF (1 year), PROMIS PH (6 months, 1 year), PROMIS MH (3 months, 6 months, and 1 year), and PROMIS PI (1 year).

### Treatment Success and Failure Rates

Mean FFU was 38.8 months (range, 14‐72 months) for the open ALR cohort and 18.2 months (range, 12‐24 months) for the arthroscopic ALR cohort. Overall, 22 open labral reconstructions (100%) were deemed successful, and 9 arthroscopic labral reconstructions (90%) were deemed successful at FFU such that there was no statistically significant difference (*P* = .313) in initial treatment failure rates. The patient who failed their labral reconstruction was a revision patient who had undergone initial labral reconstruction 52 months prior with concurrent femoral head osteochondral allograft. During the revision labral reconstruction, it was noted that the femoral head had grade 3 to 4 damage, but no additional intervention was deemed appropriate to address the femoral head cartilage loss at that time. This patient subsequently experienced failure of their revision labral repair 26 months postoperatively and underwent total hip arthroplasty.

## DISCUSSION

The results of this study suggest that open and arthroscopic ALRs with fresh meniscus allograft may significantly improve patient‐reported outcomes, but only the open cohort had significant improvements in pain at 1 year postoperatively. These results suggest that the null hypothesis can be rejected in that there were significant differences between open and arthroscopic approaches with respect to PROMs of pain through the first year after ALR performed using fresh meniscus allograft transplants. The initial success rates of 90% and 100% at mean FFUs of 18.2 and 38.8 months in arthroscopic and open approach cohort, respectively, suggest that ALR performed using fresh meniscus allografts may be safely and effectively performed using either surgical approach.

Acetabular labrum pathology impacts a significant number of patients. If labral pathology is left untreated, it often progresses to hip osteoarthritis and significant limitations on function and quality of life.^48^, ^49^, ^50^, ^51^ This concern is exacerbated for younger patients who are not ideal candidates for total hip arthroplasty. While an anatomic repair that restores the tissue's function is preferred for addressing labral deficiency, a significant proportion of labral pathology cannot be successfully repaired, and the evidence suggests that ALR is superior to labral resection for treatment of irreparable acetabular labrum deficiency.^32^, ^42^, ^52^ While there are many graft choices available for ALR, previous research at our institution reported that Missouri Osteochondral Preservation System–preserved fresh meniscus allografts are associated with clinically superior outcomes compared with fresh‐frozen tendon allograft transplants. The results from the present study provide further support for the use of high‐cell‐viability fresh meniscus allografts as a valid option for safe and effective ALR.

While ALR was initially performed using an open approach at our institution, there has been a recent shift toward an arthroscopic approach, when indicated. Although it is regarded as one of the most challenging procedures in hip arthroscopy, arthroscopic labral reconstruction has been shown to be a reasonable alternative to restore labral structure and function when labral repair is not possible. Ongoing research at this institution and many others aims to optimize surgical technique and graft choice for ALR. This study shows promise for both arthroscopic and open ALR with fresh meniscus allografts yet highlights differences in patient‐reported outcomes at the 1‐year postoperative timepoint between open and arthroscopic surgical techniques that warrant further investigation and long‐term follow‐up. Potential explanations for the differences in self‐reported pain metrics that are being investigated include patient variables (e.g., mean body mass index, pain perceptions and expectations, and activity levels), surgical technique differences (e.g., relative denervation, distraction, and capsular closure), and postoperative management protocols (e.g., restriction, physical therapy, and return‐to‐activity differences). Further research is needed to describe long‐term outcomes and compare open and arthroscopic ALRs with fresh meniscus allografts.

### Limitations

There are several limitations that should be considered when interpreting and applying the results of this study. First, this study has a relatively small sample size of 32 patients. A small sample size puts our data analysis at a high risk for encountering type II errors when comparing open versus arthroscopic approaches. Findings should be interpreted with caution, as no adjustment for multiple comparisons or baseline differences was applied due to the small cohort size. Second, this study is limited to a short‐term follow‐up of 1 year. Third, several patients in this study were also included in earlier publications from our institution reporting short‐term outcomes and comparing various hip preservation techniques. Fourth, patients undergoing open and arthroscopic ALR had 2 separate postoperative rehabilitation and limitation protocols. Because the open ALR technique included a surgical hip dislocation and greater trochanteric osteotomy, patients were kept from full weight‐bearing and range of motion until the 6‐week postoperative timepoint, 4 weeks different than what was allowed for the arthroscopic ALR cohort. While necessary, these variations in postoperative protocols may lead to unmeasurable variation in short‐term PROMs such as pain score. Fifth, there was statistically significant variability in mean body mass index, Pain Interference, and PROMIS Physical Health between the 2 treatment cohorts at the preoperative timepoint. There was also significant variability in FFU between the open ALR cohort and the arthroscopic ALR cohort. Finally, many patients underwent at least 1 concurrent procedure. While concurrent procedures were not significantly associated with failure risk, these procedures may have affected pain and recovery timelines.

## CONCLUSIONS

The results of this study suggest that open and arthroscopic ALRs with fresh meniscus allograft may significantly improve patient‐reported outcomes associated with hip function, health, and recovery at 1 year postoperatively. The open cohort, but not the arthroscopic cohort, had significant improvements in pain at 1 year postoperatively.

## DISCLOSURES

The authors (K.R., J.L.C., B.D.C., S.F.D.) declare the following financial interests/personal relationships which may be considered as potential competing interests: K.R. reports a relationship with the National Institutes of Health and Advanced Research Projects Agency for Health that includes funding grants. J.L.C. reports a relationship with Arthroscopy Association of North America, AAOS, and AO Trauma that includes funding grants; reports a relationship with Advanced Research Projects Agency for Health that includes consulting or advisory and funding grants; reports a relationship with Boehringer Ingelheim Corp USA that includes: consulting or advisory; reports a relationship with Gallant and GE Healthcare that includes funding grants; reports a relationship with Journal of Knee Surgery and Midwest Transplant Network that includes board membership; reports a relationship with Musculoskeletal Transplant Foundation Inc that includes board membership and funding grants; reports a relationship with the National Institutes of Health, OrthoBIo Therapeutic, Orthopaedic Research and Education Foundation, and Patient‐Centered Outcomes Research Institute that includes funding grants; reports a relationship with Thieme Medical Publishers and Trupanion that includes consulting or advisory; reports a relationship with Arthrex that includes consulting or advisory and funding grants. B.D.C. reports a relationship with AO Trauma North America that includes board membership; reports a relationship with Arthrex that includes funding grants; reports a relationship with CurvaFix that includes speaking and lecture fees; reports a relationship with DuPuy, A Johnson & Johnson Company that includes speaking and lecture fees; reports a relationship with Fragility Fracture Network‐USA that includes board membership; reports a relationship with Globus Medical that includes consulting or advisory; reports a relationship with International Geriatric Fracture Society, Journal of Hip Preservation, and Journal of Orthopaedic Trauma that includes board membership; reports a relationship with KCI that includes speaking and lecture fees; reports a relationship with Orthopaedic Trauma Association that includes board membership; reports a relationship with Osteocentric that includes consulting or advisory; reports a relationship with RomTech that includes equity or stocks; reports a relationship with Slack Incorporated that includes board membership; reports a relationship with Synthes that includes consulting or advisory and funding grants; reports a relationship with Urgo Medical that includes consulting or advisory. S.F.D. reports a relationship with American Orthopaedic Society for Sports Medicine that includes board membership; reports a relationship with AO North America that includes speaking and lecture fees; reports a relationship with Arthrex that includes funding grants; reports a relationship with *Arthroscopy* and Arthroscopy Association of North America that includes board membership; reports a relationship with Springer that includes consulting or advisory and funding grants; has patent pending too. The other authors (J.R.B., C.R.C.) declare that they have no known competing financial interests or personal relationships that could have appeared to influence the work reported in this article.
